# Advancements in extensive-stage small cell lung cancer therapy: from molecular profiling to the advent of precision oncology

**DOI:** 10.3389/fimmu.2025.1674449

**Published:** 2025-11-05

**Authors:** Chen Yang, Yanchi Shao, Yu Zhang, Huan Zhang, Yanbin Zhao

**Affiliations:** Department of Medical Oncology, Harbin Medical University Cancer Hospital, Harbin, Heilongjiang, China

**Keywords:** extensive-stage small-cell lung cancer, immunotherapy, molecular subtypes, new evolving targeted agents, antiangiogenic

## Abstract

Small cell lung cancer (SCLC) is challenging to manage due to its high malignancy and early metastatic spread. Although initial chemoradiotherapy responses are common, resistance rapidly develops, and long-term efficacy remains limited. Immune checkpoint inhibitors (ICIs) overcome previous survival barriers, extending overall survival (OS) and progression-free survival (PFS) in extensive-stage SCLC. Nevertheless, absolute clinical benefits remain modest. To address efficacy limitations, current research focuses on optimizing first-line strategies by exploring multimodal regimens (e.g., adding targeted therapy or radiotherapy to chemoimmunotherapy) and advancing molecular subtyping for precision oncology. Furthermore, emerging therapies such as DLL3-targeted agents, bispecific antibodies (bsAbs), antibody-drug conjugates (ADCs), and chimeric antigen receptor T-cell (CAR-T) therapy continue to demonstrate clinical progress. This review synthesizes advances in SCLC management, focusing on mechanisms and clinical applications of multimodal strategies and novel therapies. It provides guidance for clinical decisions, research directions, and survival improvement.

## 1 Introduction

Small cell lung cancer (SCLC) represents a particularly aggressive type of neuroendocrine carcinoma. This cancer is notable for its extremely rapid growth rate. Furthermore, SCLC frequently metastasizes early in its course and carries an unfavorable prognosis (1). While SCLC constitutes approximately 15% of lung cancers, it shows the most pronounced epidemiological association with smoking compared to other lung cancer subtypes (2). Globally, SCLC contributes to an estimated 200,000 annual deaths, reflecting its significant clinical burden (1, 2). SCLC staging defines limited (LS-SCLC) and extensive (ES-SCLC) disease based on metastatic spread at diagnosis. Initial diagnoses reveal ES-SCLC in approximately 70% of patients (3). ES-SCLC exhibits initial sensitivity to chemotherapy but is characterized by rapid relapse, with no significant therapeutic advances achieved prior to the immunotherapy era. Historically, the 5-year overall survival (OS) rate for ES-SCLC less than 7% (4). The advent of immunotherapy broke this plateau. In March 2019, the FDA approved atezolizumab plus platinum-etoposide chemotherapy for first-line ES-SCLC treatment, based on the Phase III IMpower133 trial (5). This marked a milestone as the first PD-L1 inhibitor approved for ES-SCLC in two decades, establishing chemoimmunotherapy as the standard first-line therapy. Later Phase III trials confirmed that adding PD-1/PD-L1 inhibitors to chemotherapy boosts survival (6–9). However, the median overall survival (mOS) with conventional chemoimmunotherapy remains limited to 12–15 months, with marginal improvements in median progression-free survival (mPFS) (10). The PFS benefit of immune checkpoint inhibitors(ICIs) exhibits a delayed-onset pattern, typically emerging after 6 months. SCLC has persistently lagged in precision oncology, necessitating novel therapeutic strategies to improve outcomes in extensive-stage disease. Recent advances in immunotherapy, targeted agents, and multimodal regimens have revolutionized the treatment landscape of ES-SCLC. This article reviews pivotal breakthroughs in first-line combination strategies, molecular subtype-guided therapy, and novel drug development for ES-SCLC. It provides a theoretical framework and practical guidance for optimizing clinical decision-making, directing future research, and improving survival outcomes.

## 2 Therapeutic advances in first-line ES-SCLC

### 2.1 First-line ES-SCLC: chemoimmunotherapy as the new standard

For nearly four decades until 2019, etoposide combined with platinum-based agents (carboplatin [EC] or cisplatin [EP]) served as the first-line standard therapy for ES-SCLC (11). The IMpower 133 trial, the first phase III study comparing chemoimmunotherapy versus chemotherapy alone in ES-SCLC, established that adding atezolizumab (PD-L1 inhibitor) to carboplatin-etoposide (EC) significantly improved outcomes. This trial met both primary endpoints: OS was prolonged (12.3 vs. 10.3 months; HR 0.70, 95% CI 0.54–0.91; *p* = 0.007), and PFS increased (5.2 vs. 4.3 months; HR 0.77, 95% CI 0.62–0.96; *p* = 0.017) (5). Notably, 5-year follow-up data revealed a 12% OS rate with chemoimmunotherapy, surpassing the historical <7% rate observed with chemotherapy alone (12). Building on IMpower133, the CASPIAN trial demonstrated significant survival benefits with first-line chemoimmunotherapy, resulting in the approval of durvalumab(PD-L1 inhibitor) for ES-SCLC (6). The CAPSTONE-1 study confirmed the substantial therapeutic advantage of adebrelimab. In this trial, the mOS achieved was 15.3 months (95% CI 13.2–17.5). This outcome surpassed the mOS benchmarks established by the IMpower 133 trial (12.3 months) and the CASPIAN study (13.0 months) (9). In contrast, PD-1 inhibitors pembrolizumab and nivolumab failed to demonstrate OS benefit (13, 14). However, Serplulimab (PD-1 inhibitor) combined with chemotherapy significantly improved OS in the ASTRUM-005 trial, achieving a mOS of 15.4 months (95% CI 13.3–NE) (8). Further validating PD-1 inhibitors, confirmatory phase III trials demonstrated efficacy of additional inhibitors: tislelizumab achieved mOS of 15.5 months in RATIONALE-312 (15), while toripalimab reached 14.6 months in EXTENTORCH (16). Other ICIs,CTLA-4 inhibitors ipilimumab (17) and tremelimumab (6) showed no significant therapeutic benefit in SCLC, whether administered alone or combined with other ICIs or chemotherapy. Consequently, atezolizumab and durvalumab combined with platinum-etoposide chemotherapy were approved by the U.S. FDA (2019) and EMA (2020) as first-line ES-SCLC therapy (18). February 2025 marked the European approval of serplulimab for first-line ES-SCLC, making it the EU’s inaugural anti-PD-1 agent for SCLC (**Table 1** summarizes the clinical trial results of first-line chemoimmunotherapy for ES-SCLC).

**Table 1:** Clinical trial results of first-line chemoimmunotherapy for ES-SCLC.

Trials	Phase	Treatment arms (n)	ORR(%)	mPFS outcomes	mOS outcomes	Grade ≥ 3 AEs (%)
IMpower 133	III	Atezolizumab+ EC(201)	60.2	5.2 months (HR = 0.77;95% CI: 0.63-0.95; P = 0.017)	12.3 months (HR= 0.70; 95% CI: 0.54-0.91; P=0.007)	58.6
		Placebo + EC (202)	64.4	4.3 months	10.3 months	57.6
CASPIAN	III	Durvalumab+EP/EC(268)	68.0	5.1 months (HR = 0.80; 95% CI: 0.66-0.96)	12.9months(HR = 0.75;95%CI: 0.62-0.91;P= 0.0032)	65.0
		Durvalumab+tremelimumab +EP/EC (268)	58.0	4.9 months (HR = 0.84; 95% CI: 0.70-1.01)	10.4months(HR = 0.82;95%CI: 0.68-1.00;P =0.045)	73.0
		EP/EC (269)	58.0	5.4 months	10.5 months	65.0
CAPSTONE-1	III	Adebrelimab + EC(230)	70.4	5.8 months (HR = 0.67; 95% CI: 0.54-0.83; P < 0.0001)	15.3months(HR = 0.72;95% CI: 0.58-0.90;P =0.0017)	85.7
		Placebo + EC (232)	65.9	5.6 months	12.8 months	84.9
KEYNOTE-604	III	Pembrolizumab+EP/EC (228)	70.6	4.5 months (HR = 0.75; 95% CI: 0.61-0.91; P = 0.0023)	10.8months(HR = 0.80;95%CI:0.64-0.98;P= 0.0164)	85.7
		Placebo + EP/EC(225)	61.8	4.3 months	9.7 months	80.3
ECOG-ACRIN EA5161	II	Nivolumab+EP/EC(80)	52.3	5.5months(HR = 0.65;95%CI: 0.46-0.91; P = 0.047)	11.3months(HR = 0.67;95%CI: 0.46-0.98;P =0.14)	77.0
		Placebo+EP/EC (80)	47.7	4.6 months	8.5 months	62.0
ASTRUM-005	III	Serplulimab+EC(389)	80.2	5.7months(HR = 0.47;95%CI: 0.38-0.59; P < 0.001)	15.4months(HR = 0.63;95%CI: 0.49-0.82;P < 0.001)	33.2
		Placebo + EC (196)	70.4	4.3 months	10.9 months	27.6
RATIONALE-312	III	Tislelizumab+EC/EP(227)	68.3	4.7months(HR = 0.64;95%CI:0.52-0.78;P< 0.001)	15.5months(HR = 0.75;95%CI: 0.61-0.93;P=0.004)	86.0
		EC/EP(230)	61.7	4.3 months	13.5 months	86.0
EXTENTORCH	III	Toripalimab+EP(223)	83.0	5.8months(HR = 0.67;95%CI:0.54–0.82;P <0.001)	14.6months(HR = 0.80;95%CI:0.65–0.98;P =0.0327)	89.6
		Placebo + EP(219)	73.5	5.6 months	13.3 months	89.4

EC, etoposide- carboplatin; EP, etoposide-cisplatin; ORR, objective response rate; PFS, progression-free survival; OS, overall survival; TRAE, treatment-related adverse even.

### 2.2 Optimizing multimodal combination therapies: synergistic efficacy through mechanism complementarity

Despite establishing a new first-line standard for ES-SCLC through the addition of ICIs to platinum-based chemotherapy, the OS benefit remains modest. Median OS is extended by only 2.0–4.7 months (6–9). Consequently, novel therapeutic approaches are urgently warranted to further enhance ES-SCLC outcomes.

#### 2.2.1 Synergistic mechanisms between anti-angiogenesis and ICIs

Targeting angiogenesis in SCLC represents a promising therapeutic strategy, given its critical role in tumor growth and chemoresistance development (19). Vascular endothelial growth factor (VEGF), a dominant pro-angiogenic factor, stimulates pathological neovascularization during the progression of this cancer (20). Elevated VEGF overexpression correlates with poor prognosis in patients with SCLC (21). In the phase II SALUTE trial, patients were randomized to receive etoposide-platinum combined with either bevacizumab(VEGFR inhibitor) or placebo, followed by maintenance therapy until disease progression or unacceptable toxicity. PFS significantly improved with bevacizumab(5.5 vs. 4.4 months; HR 0.53, 95% CI 0.32–0.86), although OS remained unchanged and treatment-related toxicity increased (22). Despite disappointing outcomes, research efforts persist in targeting SCLC angiogenesis. The limited efficacy of ICIs in SCLC is primarily attributed to its immunosuppressive and highly heterogeneous tumor microenvironment, characterized by inadequate infiltration of CD8+ T cells (23). VEGF overexpression suppresses ICAM-1 and VCAM-1 on endothelial cells, inhibiting immune cell adhesion and transendothelial migration (24). So VEGF pathway inhibition may enhance CD8+ T-cell infiltration while suppressing neointima formation, potentially augmenting anti-tumor immunity in ES-SCLC.

#### 2.2.2 The advent of four-drug combination regimens

In the phase III ETER701 study, the addition of benmelstobart (PD-L1 inhibitor) and anlotinib (VEGFR inhibitor) to EC chemotherapy markedly enhanced survival outcomes in treatment-naive ES-SCLC (25). The four-drug regimen achieved mOS of 19.3 months (HR 0.61, 95% CI 0.46-0.79) and mPFS of 6.9 months (HR 0.32), establishing a superior therapeutic option. The 2024 study of the three-agent regimen (anlotinib and chemotherapy) demonstrated a significantly prolonged mPFS (5.6 vs 4.2 months; P<0.0001) but no improvement in mOS (13.3 vs 11.9 months; P = 0.1732) versus chemotherapy alone, suggesting that the combination of ICIs, anlotinib, and chemotherapy induces synergistic enhancement of antitumor efficacy. Safety analyses revealed significantly higher rates of grade ≥3 treatment-related adverse events (TRAEs) (chemotherapy-only: 87.0% vs. three-agent: 94.3% vs. four-agent: 93.1%), TRAE-related treatment adjustments (41.9% vs. 57.4% vs. 54.9%), treatment discontinuations (4.1% vs. 8.6% vs. 11.8%), and deaths (1.6% vs. 2.5% vs. 4.5%) in the combination arms compared to chemotherapy alone. Although the ETER701 study lacked a control arm with standard chemoimmunotherapy and observed increased safety risks in multi-drug regimens, it generated valuable safety and efficacy data for first-line platinum-based chemotherapy combined with ICIs and antiangiogenic agents in ES-SCLC.

The BEAT-SC trial, the first global phase III study evaluating bevacizumab plus atezolizumab and platinum-based chemotherapy versus immune-chemotherapy control in ES-SCLC, met its primary endpoint. Preliminary analyses demonstrated significantly prolonged mPFS with bevacizumab combination therapy (5.7 vs. 4.4 months; HR = 0.70, 95% CI 0.54–0.90; *P* = 0.0060), though no mOS benefit was observed at interim analysis (26).

A pioneering February 2025 trial by Zhong Nanshan’s team revealed that first-line camrelizumab (anti-PD-1) plus apatinib (VEGFR inhibitor) and chemotherapy achieved promising efficacy with manageable toxicity in ES-SCLC. Forty previously untreated ES-SCLC patients received phased therapy in this single-arm multicenter trial. The regimen initiated with two 21-day cycles of etoposide-carboplatin (EC), then added camrelizumab and apatinib to EC for four cycles, culminating in immune-angiogenic maintenance. 94.4% of patients achieved target lesion shrinkage, with an objective response rate (ORR) of 88.9% and disease control rate (DCR) of 97.2%. The mPFS was 7.3 months, and mOS reached 17.3 months. Safety analysis revealed TRAEs in all 40 patients, with 75% experiencing grade ≥3 events, no treatment-related deaths occurred (27). Clinically, most SCLC lesions predominantly localize centrally, involving hilar regions and major vasculature (28), conferring high bleeding risk. Trials evaluating anti-angiogenic combinations(including ETER701) routinely exclude patients with major vessel invasion due to elevated bleeding risks (25). This substantial patient subset within the ES-SCLC cohort necessitates urgent intervention to bridge the therapeutic gap. Induction chemotherapy was administered before combination therapy to facilitate tumor shrinkage and perivascular separation, reducing bleeding risks from anti-VEGFR agents. This strategy further induces immunogenic cell death, augmenting the effectiveness of immunotherapy (29). In this study, all 35 evaluable patients exhibited baseline major vessel infiltration. Despite this, safety outcomes mirrored the ETER701 profile, no grade ≥3 hemoptysis or severe hemorrhage occurred (25). The regimen exhibited a manageable safety profile alongside clinically meaningful efficacy, substantiating its viability as a frontline therapeutic modality for ES-SCLC.

#### 2.2.3 Intensification of maintenance therapy

The DURABLE study (NCT04192604) was a randomized, multicenter phase 2 trial evaluating durvalumab plus anlotinib versus durvalumab alone as maintenance therapy in ES-SCLC. Compared with durvalumab monotherapy, durvalumab plus anlotinib maintenance significantly prolonged mPFS from 1.9 to 5.4 months and mOS from 12.4 to 17.4 months, with a final mOS of 20.4 versus 15.4 months (P <0.05) (30). Results indicate that durvalumab combined with anlotinib offers superior safety without compromising efficacy compared to four-drug regimens. This study proposes that incorporating anti-angiogenic agents during maintenance therapy achieves an optimized efficacy-safety balance, informing future ES-SCLC treatment strategies.

The integration of antiangiogenic agents with first-line chemoimmunotherapy may improve clinical outcomes in treatment-naïve ES-SCLC patients. However, further randomized trials and real-world evidence are required to validate this synergistic effect, while comprehensive efficacy-safety assessments remain essential for individualized therapeutic strategies.

### 2.3 Synergistic effect of radiotherapy and immunization

The limited efficacy of first-line ICI-chemotherapy combinations in ES-SCLC is compounded by predominant intrathoracic recurrences (31). This recurrence pattern underscores the potential of thoracic radiotherapy, which synergizes with ICIs through radiation-induced immunogenic cell death and enhanced tumor antigen presentation—mechanisms validated in both preclinical models and early-phase trials.

The single-arm phase II MATCH study evaluated first-line chemoimmunotherapy (IMpower133 regimen) with concurrent low-dose thoracic radiotherapy (LDRT; 15 Gy/5 fractions) in ES-SCLC. Results showed a mPFS of 6.9 months (95% CI: 5.4–9.3), representing a 1-month improvement over IMpower133, with 12-month PFS and OS rates of 27.7% and 71.9%, mOS was not reached (32). The single-arm phase II LEAD trial evaluated first-line durvalumab plus chemotherapy with concurrent LDRT in ES-SCLC, based on the CASPIAN regimen. This combination achieved a mPFS of 8.3 months, significantly exceeding the 5.1 months observed in CASPIAN, while median OS remains unreached (33).

A 2024 phase II prospective trial evaluated first-line adebrelimab plus chemotherapy followed by consolidative thoracic radiotherapy (TRT) in ES-SCLC. Combining immunotherapy with TRT significantly improved both short and long-term outcomes in ES-SCLC. For the entire cohort, mOS and PFS reached 21.4 months (95% CI: 17.2–NR) and 10.1 months (95% CI: 6.9–15.5). Treatment-associated toxicities were acceptable (grade ≥3 TRAEs: 58.2%; pneumonitis: 6%), aligning with established checkpoint inhibitor safety benchmarks. The study further demonstrated that ES-SCLC patients exhibiting ctDNA clearance or *TP53*/*RB1* co-mutation derived greater benefit from first-line immunotherapy post-radiotherapy (34).

Phase II trials demonstrate that combining radiotherapy with chemoimmunotherapy may enhance survival in SCLC. Ongoing phase III studies aim to define optimal thoracic radiotherapy parameters, including beneficiary selection, dose fractionation, and treatment timing (**Table 2** lists partial trial results of novel combination regimens for First-Line Treatment of ES-SCLC).

**Table 2:** Trials results of novel combination regimens for first-line treatment of ES-SCLC.

Trial	Phase	Treatment arms (n)	ORR(%)	mPFS outcomes	mOS outcomes	Grade≥3 AEs(%)
Chemoimmunotherapy combined with antiangiogenic agents						
ETER701	III	Benmelstobart+anlotinib+EC(246)	81.3	6.9 months(HR = 0.32;95%CI: 0.26-0.41;P < 0.001)	19.3 months(HR = 0.61;95%CI: 0.47-0.79;P < 0.001)	93.1
		Placebo + anlotinib + EC(245)	81.2	5.6 months(HR = 0.44;95%CI: 0.36-0.55;P <0.001)	13.3 months(HR = 0.86;95%CI: 0.67-1.10;P=0.1732)	94.3
		Placebo + Placebo + EC(247)	66.8	4.2 months	11.9 months	87.0
BEAT-SC	III	bevacizumab +atezolizumab+ EP/EC (166)	81.9	5.7 months(HR = 0.70;95%CI 0.54-0.90;P=0.006)	13.0 months(HR = 1.22;95%CI 0.89-1.67;P=0.221 2)	85.5
		Placebo+atezolizumab+EP/EC (164)	73.3	4.4 months	16.6 months	86.0
NCT05001412	II	camrelizumab +apatinib +EC(40)	88.9	7.3 months	17.3 months	75.0
Chemoimmunotherapy combined with radiotherapy						
MATCH	II	Atezolizumab+EC+LDRT(56)	87.5	6.9months;6-month PFS rate 56.5%;12-month PFS rate 27.7%	NR; 12-month OSrate 71.9%	91.1
LEAD	II	Durvalumab+EP/EC+LDRT(30)	87.0	8.3 months(95% CI 4.6-15.2); 6-month PFS rate 57%;12-month PFS rate 40%	NR	80.0
NCT04562337	II	Adebrelimab+EP/EC+TRT(67)	71.6	10.1months (95% CI 6.9-15.5)	21.4 months (95% CI: 17.2–NR)	58.2

EC, etoposide- carboplatin; EP, etoposide-cisplatin; ORR, objective response rate; PFS, progression-free survival; OS, overall survival; TRAE, treatment-related adverse event; NR, not reached; LDR,Tlow-dose thoracic radiotherapy; TRT,thoracic radiotherapy.

## 3 Molecular subtype-driven precision therapeutics

Historically, SCLC was defined as a uniform entity characterized by near-ubiquitous inactivation of *TP53* and *RB1* tumor suppressors, high proliferation rates, and elevated tumor mutational burden (TMB) (35). However, emerging evidence of molecular heterogeneity has shifted research focus toward identifying predictive biomarkers and distinct transcriptional subtypes with differential therapeutic vulnerabilities.

Immunohistochemical profiling of mouse models identified distinct molecular subtypes defined by lineage-specific transcription factors (36). The SCLC-A is defined by high expression of the neuroendocrine transcription factor achaete-scute homolog 1 (*ASCL1*), with concomitant upregulation of *MYCL*, *BCL2*, *SOX2*, and *DLL3* (delta-like ligand 3).SCLC-N, marked by *NEUROD1*-driven neuroendocrine differentiation, co-expresses oncogenic *MYC* with neural development factors *INSM1* and *HES6*. SCLC-P is governed by the lineage-specific factor *POU2F3*, whereas SCLC-Y demonstrates *YAP1*-mediated Hippo pathway dysregulation. Further immunohistochemical analysis revealed uniformly low *YAP1* expression across all subtypes, precluding definitive identification of the SCLC-Y subgroup (37).

A distinct subtype designated SCLC-I (inflamed) was subsequently identified, characterized by the absence of *ASCL1*, *NEUROD1*, and *POU2F3* expression but elevated infiltration of immune cells (T cells, NK cells, macrophages). This subtype also exhibited overexpression of *HLA* molecules, immune checkpoints (e.g., *PD-1*, *PD-L1*, *CTLA4*, *CD38*, *IDO1*, *TIGIT*, *VISTA*, *ICOS*, *LAG3*), and chemokines (e.g., *CCL5*, *CXCL10*) compared to other subtypes (38). The neuroendocrine markers chromogranin A (*CHGA*) and synaptophysin (*SYP*) were highly expressed in SCLC-A and SCLC-N, whereas RE1-silencing transcription factor (*REST*), a repressor of neuroendocrine differentiation, was elevated in SCLC-P and SCLC-I (39). Molecular subtyping of SCLC guides personalized therapy, with the SCLC-I subtype showing preferential response to ICI. In the IMpower133 trial, chemotherapy combined with atezolizumab improved outcomes across subtypes, but the most significant overall survival benefit was observed in SCLC-I (mOS 18.2 vs. 10.4 months; HR = 0.62, p<0.01) (38). Preclinical and clinical data indicate distinct therapeutic vulnerabilities across SCLC subtypes: SCLC-A exhibits variable sensitivity to platinum-based agents, alongside potential responses to *BCL2* inhibitors and *DLL3*-targeted therapies. SCLC-N demonstrates platinum resistance but high susceptibility to Aurora kinase inhibitors (AURK inhibitors) and PARP inhibitors. SCLC-P shows responsiveness to PARP inhibitors and antimetabolites. Both SCLC-P and SCLC-N demonstrated high sensitivity to AURK inhibitors, driven by convergent *MYC* amplification. Intratumoral heterogeneity was further evidenced by single-cell co-expression of lineage transcription factors, suggesting phenotypic plasticity (38, 40, 41). Ireland et al. demonstrated that MYC activates Notch signaling to drive a subtype shift in SCLC subtypes from *ASCL1* through *NEUROD1 to YAP1* states, facilitating dedifferentiation from neuroendocrine (NE) to non-neuroendocrine (non-NE) phenotypes (41). Gay et al. demonstrated that cisplatin induces subtype switching from platinum-sensitive SCLC-A to platinum-resistant SCLC-I in cell line and xenograft (CDX)models, establishing this transition as a key mediator of acquired resistance. The resulting SCLC-I subtype exhibits stem cell-like plasticity and enhanced immunotherapy sensitivity (38).

Recent studies identify *HMGB3* and *CASP1* as prognostic biomarkers in SCLC. Additionally, *ZFHX3* mutation predicts immune-hot tumor development and indicates potential benefit from ICIs (42). In this investigation, Non-negative matrix factorization (NMF) classified tumors from 112 treatment-naïve patients into four subtypes (NMF1–4). The NMF1 subtype exhibited hyperproliferation, replication stress, and neuroendocrine differentiation, whereas NMF2 showed elevated *DLL3* expression, suggesting susceptibility to DLL3-targeted therapy. The NMF3 subtype exhibits an elevated epithelial-mesenchymal transition (EMT) signature and hyperactivated receptor tyrosine kinase (*RTK*) pathways, suggesting potential vulnerability to RTK-targeted inhibitors (43). NMF4 exhibits non-neuroendocrine features with high *MYC* and *POU2F3* expression, and demonstrates sensitivity to AURK inhibitor. both NMF3 and NMF4 show elevated immune infiltration scores (44). These multi-omics subtypes thus offer clinically actionable biomarkers for personalized SCLC treatment.

Integrating subtype-directed targeted therapies with dynamic modulation of phenotypic plasticity constitutes a cornerstone of precision oncology in SCLC, addressing acquired resistance through controlled subtype evolution. However, whether molecular subtype differences correlate with tumor stage, metastatic potential, tissue specificity, or immune microenvironment remains unverified. Further research is thus imperative to translate SCLC molecular subtyping into clinical practice.

## 4 Advances in novel drug development

### 4.1 *DLL3* targeted therapy

*DLL3* inhibits Notch signaling to drive SCLC proliferation through Snail activation. Its knockdown suppresses tumor growth, while overexpression accelerates proliferation (45). *DLL3* knockdown inhibits tumor proliferation, while its overexpression enhances proliferation. While *DLL3* expression is confined to intracellular compartments in normal tissues, 85% of SCLC cases exhibit prominent surface overexpression (46). The limited efficacy of ICIs in SCLC may stem from DLL3-mediated immunosuppression. High *DLL3* expression correlates with suppression of immune-related pathways and impaired dendritic cell function, fostering an immunosuppressive microenvironment. In this cohort, elevated *DLL3* levels predicted poorer PFS in patients receiving platinum-etoposide combined with anti-PD-L1 therapy (47). Consequently, *DLL3* represents a promising therapeutic target for SCLC, offering new avenues to combat this aggressive malignancy with dismal prognosis.

**Figure 1:**
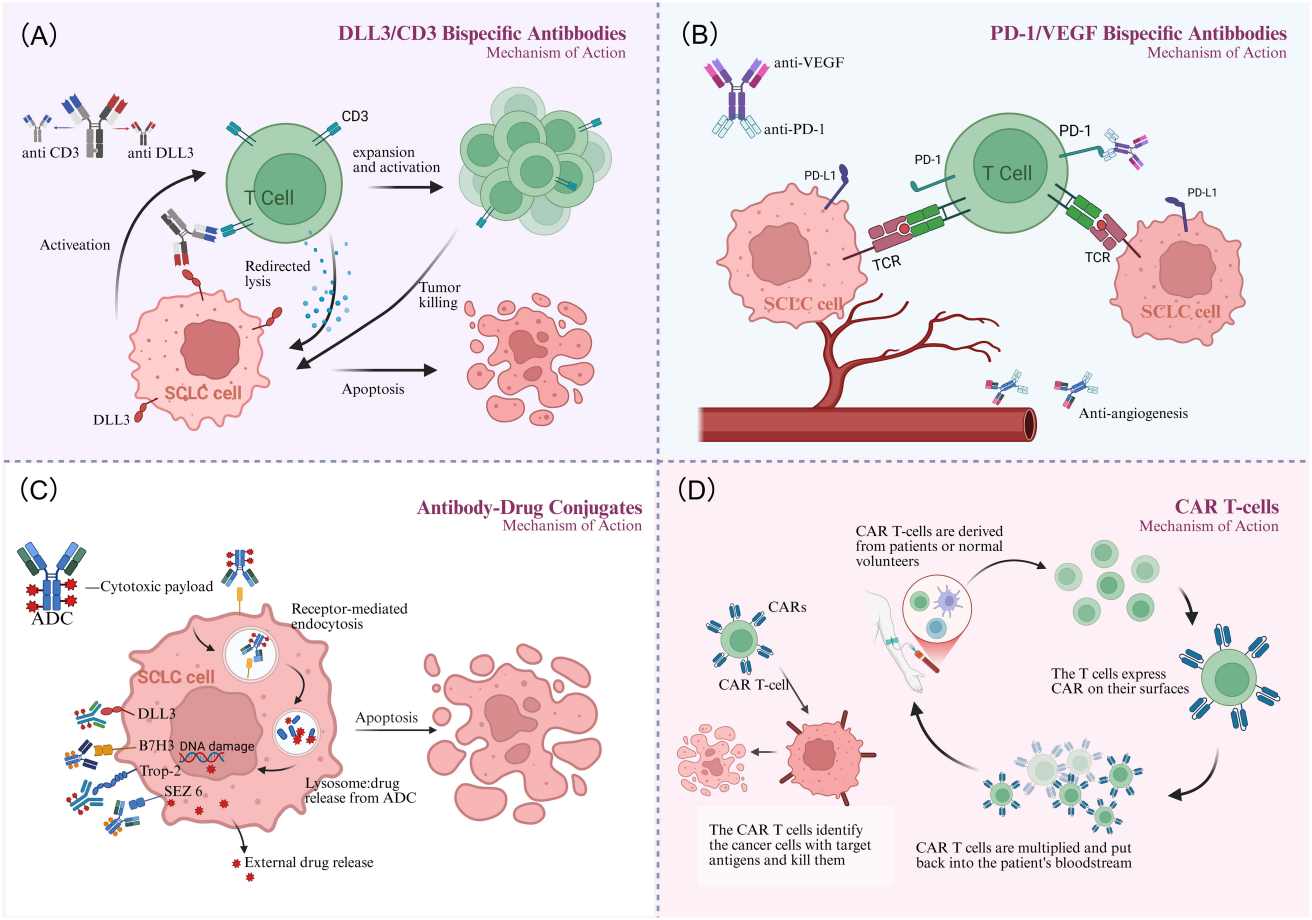
Mechanism of action. **(A)** The DLL3/CD3 bispecific antibody bridges T cells to tumor cells, activating T-cell-mediated cytotoxicity and leading to targeted tumor cell lysis. **(B)** The PD-1/VEGF bispecific antibody synergistically inhibits tumor growth via dual mechanisms: reversing T-cell suppression and inhibiting tumor angiogenesis. **(C)** Antibody-drug conjugates (ADCs) bind to tumor surface antigens, are internalized, and release their cytotoxic payload within lysosomes to kill the cancer cell. **(D)** CAR-T therapy engineers a patient's T cells to recognize and kill tumor cells upon reinfusion. Created in http://BioRender.com.

As a bispecific T-cell engager (BiTE), Tarlatamab mediates tumor lysis by bridging DLL3-expressing cancer cells with CD3-positive T lymphocytes, thereby activating cytotoxic T-cell responses against SCLC tumors(**Figure 1A**). In the phase I DeLLphi-300 trial (NCT03319940), tarlatamab yielded a 23.4% objective response rate, 13.2-month median overall survival, and 3.7-month median progression-free survival in heavily pretreated SCLC patients (48). Cytokine release syndrome(CRS) was the most frequent TRAE (52%) but was typically low-grade (49). On May 16, 2024, the FDA granted accelerated approval to tarlatamab for ES-SCLC with progression during or after platinum-based chemotherapy, establishing it as the first BiTE therapy approved for SCLC (50). This approval was supported by the Phase II DeLLphi-301 trial (NCT05361395), an open-label study in relapsed/refractory SCLC patients progressing after ≥2 prior therapies. Tarlatamab 10 mg and 100 mg cohorts both demonstrated durable responses and manageable safety (51). Extended follow-up revealed differentiated outcomes the 10 mg arm showed significantly higher ORR (40% vs 32%; P = 0.03), comparable mPFS (4.3 vs 3.9 mo; HR = 0.91), and superior mOS (14.3 mo vs not reached; HR = 0.72). Grade≥3 TRAEs occurred in 29% (10 mg) vs 52% (100 mg), establishing 10 mg as the optimal dosing strategy (52). Although the DeLLphi-301 trial yielded promising results, its lack of a standard treatment control group limits interpretability. This gap is being addressed by the ongoing phase III DeLLphi-304 trial (NCT05740566), comparing tarlatamab (10 mg biweekly) with standard therapy. Meanwhile, the phase Ib DeLLphi-303 study evaluated tarlatamab plus a PD-L1 inhibitor as maintenance therapy after first-line chemoimmunotherapy in ES-SCLC. Preliminary 2024 data demonstrated a DCR of 62.5% for this combination.The9-month OS rates were 91.8% (tarlatamab plus durvalumab) and 86.7% (tarlatamab plus atezolizumab), with mPFS of 5.3 and 5.6 months, respectively, significantly surpassing PD-L1 inhibitor monotherapy in both PFS and OS (53). Based on these results, tarlatamab has emerged as a pivotal ES-SCLC therapy, justifying expanded research into combinatorial strategies for superior clinical outcomes. MK-6070 is a trispecific T-cell engager targeting DLL3, CD3, and albumin. The ongoing phase I/II trial (NCT04471727) evaluates its safety and efficacy alone or combined with atezolizumab in advanced DLL3-positive tumors. Interim analysis confirmed robust anti-tumor responses in refractory SCLC, including those with baseline brain metastases (ORR: 37%) (54).

### 4.2 PD-1/L1 and VEGF bispecific antibodies

Bispecific antibodies co-targeting immune checkpoints and angiogenic pathways have emerged as promising therapeutic strategies for SCLC (**Figure 1B**), enabling more effective remodeling of the immunosuppressive tumor microenvironment (TME). A phase II study (ChiCTR2200059911) combines the PD-L1/VEGF-A bispecific antibody PM8002 with paclitaxel in advanced SCLC. Patients progressing after first-line platinum-based chemotherapy achieved an ORR of 72.7%, a DCR of 81.8%, and a mPFS of 5.5 months (95% CI: 2.8–NR). Prior immunotherapy-treated patients attained an ORR of 42.9%, suggesting potential to overcome immunotherapy resistance (55). A phase III trial (NCT06616532) is currently evaluating PM8002 combined with paclitaxel versus standard chemotherapy in relapsed SCLC. Additionally, phase II data presented at the 2025 European Lung Cancer Congress demonstrated that first-line PM8002 combined with etoposide and carboplatin in ES-SCLC achieved an unconfirmed ORR of 87.5%, a confirmed ORR of 85.4%, and a DCR of 97.9%. The mPFS was 6.9 months, with a 12-month OS rate of 72.7%. These results significantly surpassed those of existing PD-L1 monoclonal antibody-based combination chemotherapy regimens (56). A global phase III multicenter study (NCT06712355) is investigating first-line atezolizumab plus chemotherapy versus chemotherapy alone.

AK112, a bispecific antibody targeting PD-1 and VEGF-A, demonstrated favorable safety and preliminary efficacy in a phase Ib trial evaluating its combination with chemotherapy as first-line treatment for ES-SCLC. The ORR was 80% (16/20), with the 10 mg/kg cohort showing optimal activity (ORR 90.9%, 10/11) and mPFS of 6.9 months, OS data remain immature (57). Phase II trials assessing AK112 plus chemotherapy in first-line ES-SCLC, and AK112 combined with irinotecan liposome in second-line SCLC, are currently in development. If phase III data confirm survival benefits, dual PD-1/L1 and VEGF blockade may replace PD-L1 monoclonal antibodies as the new frontline standard for SCLC, while providing a high-response-rate, chemotherapy-free option for second-line therapy.

### 4.3 Antibody-drug conjugates

Antibody-drug conjugates (ADCs) (**Figure 1C**) have emerged as a promising therapeutic strategy for SCLC (58). As an immune checkpoint molecule, B7H3 is aberrantly expressed in 65% of SCLC tumors, where its overexpression drives disease progression and predicts poor survival, thereby nominating it as a promising immunotherapeutic target (59–61). I-DXd is a B7H3-targeted ADC comprising an anti-B7H3 monoclonal antibody linked to a topoisomerase I inhibitor via a tetrapeptide-based cleavable linker. In the phase I/II DS7300-A-J101 trial, the I-DXd showed promising antitumor activity and acceptable toxicity in advanced solid tumors. Among 22 patients with pretreated ES-SCLC, the ORR was 52.4%, with mPFS of 5.6 months and mOS of 12.2 months (62). Dose optimization analysis of the phase II IDeate-Lung01 trial established 12 mg/kg as the recommended dose for I-DXd in ES-SCLC, based on a balanced efficacy-safety profile. Key outcomes included ORR 54.8%, mPFS 5.5 months, and mOS 11.8 months, alongside significant intracranial disease control. These results establish I-DXd as a promising therapeutic option for ES-SCLC and support ongoing investigations into novel I-DXd-based combination strategies.

HS-20093, a B7-H3-targeted ADC independently developed in China, demonstrated robust clinical efficacy in a phase I trial involving patients with ES-SCLC previously treated with platinum-based chemotherapy with or without ICIs. At doses of 8 mg/kg and 10 mg/kg, the ORR were 61.3% and 50.0%, with mPFS of 5.9 months and 7.3 months. The 8 mg/kg dose exhibited a more favorable safety profile, and no significant correlation was observed between B7-H3 expression levels and antitumor activity (63). A phase III trial (NCT06498479) comparing HS-20093 with standard second-line therapy in ES-SCLC is currently underway.

Trophoblast cell surface antigen 2 (TROP2), a transmembrane glycoprotein overexpressed in diverse solid tumors, drives tumorigenesis, invasion, and metastasis, establishing it as a promising therapeutic target. Sacituzumab Govitecan (SG), a TROP2-directed ADC, has demonstrated clinical efficacy in breast cancer (64). The phase II TROPiCS-03 trial (NCT03964727) evaluated SG in 43 ES-SCLC patients progressing after first-line platinum-based chemotherapy and immunotherapy. Published 2024 results revealed an ORR of 41.9%, with mPFS of 4.40 months, and mOS of 13.60 months. Notably, SG exhibited antitumor activity in both platinum-resistant (ORR 35.0%) and platinum-sensitive (ORR 47.8%) subgroups (65).

Emerging ADCs targeting novel antigens show therapeutic potential. Seizure-related homolog 6 (SEZ6), a transmembrane glycoprotein overexpressed in SCLC but minimally detectable in normal tissues, demonstrates high tumor specificity (66). SEZ6-directed ADCs represent an emerging therapeutic strategy for SCLC. ABBV-706, an investigational ADC targeting SEZ6 conjugated to a novel topoisomerase I inhibitor payload, is being evaluated in a phase I trial (NCT05599984). This study assesses ABBV-706 as monotherapy and in combination with the PD-1 inhibitor budigalimab (ABBV-181), carboplatin, or cisplatin in advanced SCLC and other neuroendocrine tumors (NETs) (67). Preliminary results from the dose-escalation cohort of ABBV-706’s first-in-human trial, presented at the May 2024 ASCO Annual Meeting, reported a confirmed overall ORR of 43.8% (21/48) in efficacy-evaluable patients, with the SCLC subgroup achieving an ORR of 60.9% (14/23). TRAEs of ≥ grade 3 included neutropenia (42%), anemia (42%), and leukopenia (28%). In October 2024, the U.S. FDA granted ABBV-706 orphan drug designation for SCLC, supporting its therapeutic potential for this aggressive malignancy and validating SEZ6 as a promising target.

ZL-1310 is a DLL3-targeted ADC. Global Phase Ia data (NCT06179069) presented at the 2024 European Neuroendocrine Tumor Association Congress (ENETS) demonstrated an ORR of 74% across all tested dose levels in ES-SCLC (68). Moreover, ZL-1310 exhibited significant blood-brain barrier penetration, achieving 100% intracranial tumor regression in patients with brain metastases.

### 4.4 Cell therapy

Chimeric Antigen Receptor T-cell(CAR-T) therapy is an adoptive immunotherapy that involves extracting autologous T cells, genetically engineering them to express tumor-specific antigen receptors, and reinfusing these modified cells to eliminate malignant cells (**Figure 1D**). CAR-T therapy has demonstrated efficacy in hematologic malignancies (e.g., B-cell non-Hodgkin lymphoma and multiple myeloma) (69). However, its application to solid tumors faces challenges including an TME and lack of ideal targets. In SCLC, bone marrow infiltration by tumor cells and high circulating tumor cell counts may render it amenable to CAR-T therapy (70). The high surface expression of DLL3 on tumor cells renders it an ideal therapeutic target. Zhang et al. developed allogeneic CAR-T cells targeting DLL3. These cells were genetically edited to partially remove the T-cell receptor (TCR), thereby minimizing rejection risk, and incorporated a safety switch to control potential toxicity. Both *in vitro* experiments and murine models demonstrated potent tumor-killing efficacy with low toxicity (71). The first clinical trial evaluating DLL3-targeted CAR-T therapy (NCT05680922) is currently enrolling extensively pretreated ES-SCLC patients across multiple U.S. centers. Preliminary data demonstrate initial safety and efficacy feasibility in this population refractory to standard platinum-etoposide-atezolizumab regimens.

Ganglioside GD2 represents another promising target for CAR-T therapy, being widely expressed in neural stem cells. The disialylated variant demonstrates minimal presence in healthy tissues but becomes highly enriched in tumor microenvironments, establishing its candidacy as a selective therapeutic target (72). A phase I open-label trial (NCT05620342) is currently evaluating the safety and preliminary efficacy of iC9-GD2.CAR.IL-15 T cells in up to 24 patients with platinum-refractory or PD-L1 inhibitor-resistant ES-SCLC or stage IV NSCLC.

Despite challenges in cost and toxicity management, CAR-T therapy demonstrates promising long-term potential, exhibiting durable antitumor responses in preclinical models and achieving sustained remission without repeated infusions in clinical cases. These advances position CAR-T as a viable therapeutic strategy for SCLC (**Table 3** displays selected clinical trials of new drugs for ES-SCLC).

**Table 3:** Selected clinical trials of new drugs for ES-SCLC.

Properties	Drug	Trial identifier	Phase	Indications	Treatment arms
DLL3/CD3 BiTE	tarlatamab	NCT03319940 (DeLLphi-300)	I	relapsed SCLC	tarlatamab
		NCT05060016 (DeLLphi-301)	II	relapsed SCLC	Tarlatamab (10mg)
					Tarlatamab (100mg)
		NCT05740566 (DeLLphi-304)	III	progressed after first-line platinum-based chemotherapy in ES-SCLC	Tarlatamab
					standard therapy
		NCT05361395 (DeLLphi-303)	Ib	maintenance therapy after first-line chemoimmunotherapy for ES-SCLC	Tarlatamab+Atezolizumab
					Tarlatamab+Durvalumab
DLL3/CD3/albumin TriTE	MK-6070	NCT04471727	I/II	advanced DLL3-positive tumors	MK-6070
PD-1/L1 and VEGF Bispecific Antibodies	PM8002	ChiCTR2200059911	II	progressed after first-line platinum-based chemotherapy in ES-SCLC	PM8002 + paclitaxel
		NCT06616532	III	progressed after first-line platinum-based chemotherapy in ES-SCLC	PM8002 + paclitaxel
					standard chemotherapy
		NCT05844150	II	first-line treatment for ES-SCLC	PM8002+EC
		NCT06712355	III	first-line treatment for ES-SCLC	atezolizumab +EC
					PM8002+EC
	AK112	NCT05116007	Ib	first-line treatment for ES-SCLC	AK112+EC
		NCT07057791	II	first-line treatment for ES-SCLC	AK112+EC
		NCT06478043	II	second-line treatment for ES-SCLC	AK112+irinotecan liposome
B7H3-targeted ADC	I-DXd	NCT04145622 (DS7300-A-J101)	I/II	advanced solid tumors	I-DXd
		NCT05280470 (IDeate-Lung01)	II	pretreated ES-SCLC	I-DXd
	HS-20093	NCT05276609	I	pretreated ES-SCLC	HS-20093 (8 mg/kg)
					HS-20093 (10 mg/kg)
		NCT06498479	III	relapsed SCLC	HS-20093
					Topotecan
TROP2-targeted ADC	SG	NCT03964727	II	metastatic Solid Tumors	SG
SEZ6-targeted ADC	ABBV-706	NCT05599984	I	advanced solid tumors	ABBV-706
DLL3-targeted ADC	ZL-1310	NCT06179069	Ia	ES-SCLC	ZL-1310
DLL3-targeted CAR-T	LB2102	NCT05680922	I	pretreated ES-SCLC	LB2102
GD 2-targeted CAR-T	iC9.GD2.CAR.IL-15 T	NCT 05620342	I	platinum-refractory and/or PD-L1 inhibitor-resistant ES-SCLC or IV NSCLC	iC9.GD2.CAR.IL-15 T

EC, etoposide- carboplatin; BiTE, bispecific T-cell engager; TriTE, trispecific T-cell engager; ADC, antibody-drug conjugate; CAR-T, chimeric antigen receptor T-cell.

## 5 The impact of treatment on patient quality of life

Contemporary therapeutic strategies for ES-SCLC increasingly prioritize both survival benefit and patient quality of life (QoL). As the standard first-line regimen, chemoimmunotherapy significantly improve overall survival. This combination also provides durable responses and a manageable toxicity profile, which help alleviate tumor-related symptoms like cough and dyspnea (10). The potential for extended treatment-free intervals may better preserve patients’ daily functional capacity. Even with more intensive four-drug regimens, a high DCR and significant symptom relief are achievable. The main adverse reactions are typically predictable and manageable with supportive care, and do not significantly increase treatment-related mortality (25–27). Consequently, patients can achieve prolonged survival while maintaining a relatively stable QoL. Novel therapeutic agents offer new options for patients with refractory disease. These agents demonstrate promising efficacy while exhibiting distinct toxicity profiles compared to traditional chemotherapy. Some regimens are potentially more manageable, which may help patients maintain their QoL during subsequent treatment phases.

Ultimately, successful supportive care, close monitoring and timely management of adverse reactions, along with multidisciplinary, individualized treatment strategies, are key to maximizing therapeutic efficacy and minimizing the negative impact on QoL.

## 6 Future directions and prospects

Recent therapeutic advances for ES-SCLC have yielded diverse breakthroughs, creating both significant clinical opportunities and challenges. However, limitations persist, including the predominance of single-arm trials and discrepancies between clinical trial populations and real-world cohorts. Thus, clinical findings require validation through rigorously designed head-to-head trials, systematic reviews with network meta-analysis, and real-world evidence studies that capture longitudinal treatment patterns. Current immune-targeted therapies in SCLC are constrained by molecular heterogeneity, wherein distinct molecular subtypes exhibit differential therapeutic vulnerabilities (73). Future studies should prioritize biomarker-guided patient stratification to optimize treatment regimens. Moreover, whole-genome sequencing (WGS) and integrated multi-omics approaches (74)—spanning genomics, transcriptomic, epigenomics, and metabolomics—are essential to elucidate mechanisms underlying SCLC pathogenesis, plasticity, metastasis, and therapy resistance. By integrating WGS data with transcriptomic and epigenomic profiles, it is possible to reconstruct regulatory networks linking genetic alterations to functional phenotypes, thereby clarifying the molecular mechanisms underlying phenotypic transformation, metastatic progression, and drug resistance. At the clinical level, multi-omics analysis facilitates the identification of novel therapeutic targets, improves prediction of drug responses, and elucidates resistance mechanisms. Ultimately, these approaches support the development of personalized treatment strategies based on individual tumor molecular maps, advancing SCLC care toward precision medicine. However, a critical barrier in SCLC research remains the scarcity of treatment-naïve and post-progression tissue samples. Beyond liquid biopsies (75), patient-derived xenografts (PDX) (76) and rapid autopsy collections serve as viable alternatives. Concurrently, preclinical models—encompassing murine cell lines, organoids (77), PDX, and genetically engineered mouse models (GEMMs)—have undergone significant refinement. These systems are essential for bridging fundamental discoveries to clinical implementation, while concurrently serving as cornerstones for innovative therapeutic development.

## 7 Summarization

This review synthesizes recent advances in ES-SCLC management, encompassing breakthroughs in first-line combination strategies, molecular subtype-guided therapies, and novel agent development. However, current salvage therapies offer limited efficacy, with survival benefits primarily derived from initial treatment. Future research should prioritize combination regimens and agents targeting novel mechanisms to overcome therapeutic barriers.
